# Shotgun Lipidomics for the Determination of Phospholipid and Eicosanoid Profiles in Atlantic Salmon (*Salmo salar* L.) Muscle Tissue Using Electrospray Ionization (ESI)-MS/MS Spectrometric Analysis

**DOI:** 10.3390/ijms22052272

**Published:** 2021-02-25

**Authors:** JuDong Yeo, Christopher C. Parrish

**Affiliations:** Department of Ocean Sciences, Memorial University of Newfoundland, St. John’s, NL A1C 5S7, Canada; cparrish@mun.ca

**Keywords:** eicosanoid, MS/MS, phospholipids, salmon, lipidomics

## Abstract

Shotgun lipidomics was applied to identify and quantify phospholipids (PLs) in salmon muscle tissue by focusing on the distribution of ω-3 fatty acids (e.g., docosahexaenoic acid (DHA) and eicosapentaenoic acid (EPA)) in the form of phospholipids, as well as to identify and quantify eicosanoids, which has not yet been attempted in Atlantic salmon muscle. Shotgun lipidomics enabled the identification of 43 PL species belonging to four different classes: phosphatidylcholines (PCs), phosphatidylethanolamines (PEs), phosphatidylserines (PSs), and phosphatidylinositols (PIs). Among others, 16:0-22:6 PtdCho *m/z* [M + Na]^+^ at 828.4 was the predominant PL species in salmon muscle tissue. The present study provided the quantification of individual phospholipid species, which has not been performed for salmon muscle tissue so far. In addition, two eicosanoids—prostaglandin E2 (PGE2) and prostaglandin F3α (PGF3α)—were identified for the first time in salmon muscle. Thus, the rapid and high-throughput shotgun lipidomics approach should shed new light on phospholipids and eicosanoids in salmon muscle tissue.

## 1. Introduction

Many epidemiological studies have reported that the consumption of ω3 polyunsaturated fatty acids (PUFAs) suppresses chronic diseases such as asthma, cystic fibrosis, rheumatoid arthritis, behavioural disorders, and cardiovascular disease (CVD), as well as improving brain development and function [1]. Salmon muscle tissue has an abundance of ω3 PUFAs, including eicosapentaenoic acid (20:5ω3, EPA) and docosahexaenoic acid (22:6ω3, DHA); thus, it is recommended as an excellent food for supplying those PUFAs. The phospholipids in fish contain a high proportion of ω3 PUFAs. Özdemir et al. [2] reported that 33.1%–50.1% of ω3 PUFAs in six different fish taxa from the Northwest Atlantic were in phospholipids (PLs).

Phospholipids account for approximately 60 mol% of the lipid mass in eukaryotic cells and play a crucial role in sustaining cell life [3]. The amphiphilic structure of phospholipids consists of at least one hydrophilic phosphate group and two (or sometimes one) lipophilic aliphatic chains. This structure is responsible for the construction of the cell membrane by forming a lipid bilayer that contributes to membrane fusion and arachidonic acid (ARA) secretion and provides protection from oxidation [4,5,6,7]. Phosphatidylcholine (PC) and phosphatidylethanolamine (PE) are the dominant phospholipids in most eukaryotic membranes. They are usually present asymmetrically in membranes; PC is mainly found in the outer membrane, whereas PE and phosphatidylserine (PS) are mainly present in the inner membrane [8].

There have been several attempts to characterize phospholipids in salmon muscle tissue. Peng et al. [9] isolated phospholipid from the lipid extract of Atlantic salmon fry and measured their total phospholipid content and fatty acid profiles. Haq and Chun [10] found four PL groups: PC, PE, PS, and PI (phosphatidylinositol) in the by-products of Atlantic salmon using high-performance liquid chromatography (HPLC) equipped with an evaporative light-scattering detector (ELSD). These works provided valuable information on the potential quantification and determination of the major phospholipid groups in salmon muscle tissue; however, they revealed limitations in the identification and quantification of individual phospholipids, since the approaches used do not offer detailed information on the phosphate head group and two lipophilic aliphatic chains of each phospholipid.

Shotgun lipidomics, a direct infusion into the mass spectrometer system, has been employed for the characterization of a wide range of lipid species. An important advantage of shotgun lipidomics is that a mass spectrum of samples can be obtained at a constant concentration of the solution (target compounds) during direct infusion [11]. This system provides unlimited time to use multiple fragmentation strategies such as precursor-ion, product-ion, and neutral loss scanning modes and selected reaction monitoring (SRM) during direct infusion. This facilitates the identification of the different classes of lipids by providing unlimited opportunities to attempt multiple fragmentation modes and the adjustment of mass analysis conditions such as the ionization condition, gas pressures, collision energies, etc., which are restricted in the HPLC-MS/MS system due to one-time injection and limited retention/running time. On the other hand, there is a limitation in the use of shotgun-based mass spectrometric analysis; namely, a direct infusion of samples to the mass spectrometer without physical separation by a HPLC system causes an interruption in the quantitative analysis of certain molecules due to isotope overlap. For instance, the M + 2 isotope of TAG 54:4 shows the same nominal mass as TAG 54:3, resulting in interference in the quantification of neighbouring molecules, which can be addressed by an HPLC-MS system. Moreover, the lack of crucial information such as retention time, which can be acquired in an HPLC system, is another limitation of shotgun-based mass spectrometric analysis in the identification process.

Electrospray ionization (ESI) mass spectrometry was first applied to the identification of the glycerophosphocholine, known as lipid platelet-activating factor (PAF), and since then it has been widely used for the analysis of phospholipids in a variety of biological samples [12]. ESI is a soft ionization technique that generates minimal and selective ion-source fragmentation under optimal analysis conditions. In tandem mass spectrometry (multiple mass analyzers such as MS/MS), the first analyzer is assigned to the selection of precursor ions after the ionization process. The selected precursor ions are subsequently accelerated by an electronic potential and enter the collision cells for the fragmentation process through collision-induced dissociation (CID), followed by the detection of generated product ions in the second analyzer [3]. There are four primary MS/MS approaches: product-ion analysis mode, precursor-ion scanning, neutral loss (NL) scanning, and selected reaction monitoring (SRM) mode. These various MS/MS scanning modes facilitate the identification of a wide spectrum of molecules that possess different ionization patterns. Meanwhile, high-resolution mass spectrometry (HRMS) has also been effectively employed for lipidomics. For example, Marqueño et al. [13] utilized HPLC-HRMS for the first time for the determination of lipid species in the muscle of two fish species: *Barbus meridionalis* and *Squalius laietanus*. So far, much of the research on the identification of phospholipids has been carried out using the product-ion analysis mode, which utilizes fragment ions to identify each phospholipid. However, this mode is complicated, and it is a challenge to confirm all the constituents of phospholipids, such as two fatty acid moieties and the phosphate group, by using only acquired fragment ions. On the other hand, the neutral loss (NL) scanning mode facilitates the confirmation of those fatty acids and the phosphate group of phospholipids by designating the desired neutral loss (i.e., NL 256 for palmitic acid, NL 278 for linolenic acid, NL 183 for phosphocholine, etc.) of the target molecules, leading to an increase in efficiency and accuracy in the identification of phospholipids. Thus, it is expected that the application of the neutral loss (NL) scanning mode will improve the identification process of phospholipids in marine taxa. Indeed, neutral loss (NL) scanning was an effective means for the identification and quantification of phospholipids in marine species such as mussels, clams, and whelks [13,14,15,16].

Prostaglandins have been referred to as ‘local hormones’ and are responsible for intercellular signaling, sustaining homeostatic functions, and mediating pathogenic mechanisms [17]. Eicosanoids, a sub-category of oxylipins, are derived by the enzymatic or non-enzymatic oxidation of arachidonic acid or other PUFAs [18]. Eicosanoids are involved in a wide range of nervous system diseases such as inflammation, neurodegenerative diseases, cancer, central nervous system injury, and neuropsychiatric conditions [19,20,21,22,23]. Until now, eicosanoids have not been identified and quantified in Atlantic salmon muscle. Thus, the advanced shotgun lipidomics approach would be a useful method for exploring eicosanoids in marine fauna.

The objective of this study was to employ shotgun lipidomics using ESI-MS/MS, along with various scanning modes, for the identification and quantification of phospholipid and eicosanoid profiles in lipid extracts of salmon muscle tissue.

## 2. Results

### 2.1. Identification of Phosphatidylcholine (PC) in Salmon Muscle Tissue by Neutral Loss (NL) Scanning in the Positive Mode

The identification of individual PCs in salmon muscle tissue was carried out by applying NL scanning in the positive mode. In the present study, prior to the injection of sample solutions to the mass spectrometer, sodium adduction was carried out in order to strengthen ion intensity of molecules, which improves the detection of target compounds. Sodium adduction was preferred to other methods such as lithium and ammonium adduction since sodium adduction has been widely used for the identification and quantification of phospholipids in biological samples and this facilitates easy comparison among results from different studies. Moreover, neutral loss scanning was employed to scan the phosphocholine head group. In the preliminary test, neutral loss scanning mode showed much higher intensity compared to the precursor ion scanning mode, leading to the use of neutral loss scanning for the analysis of PC. NL scanning mode contributes greatly to detecting molecules that possess specific fragments of lipids such as *sn*-1,2 fatty acids and polar head groups of phospholipids (i.e., NL183.1 for phosphocholine and NL 59.0 for trimethylamine in the positive ion mode).

**Figure 1:**
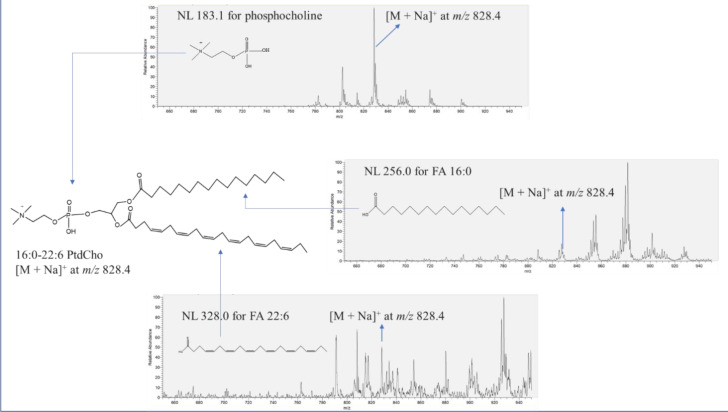
Identification of 16:0-22:6 PtdCho ([M + Na]^+^ at *m/z* 828.4) in salmon muscle tissue by neutral loss (NL) scanning in the positive mode. NL 183.1, 256.0, and 328.0 provide information on the presence of phosphocholine, FA 16:0 (palmitic acid), and FA 22:6 (docosahexaenoic acid) moieties of the structure of 16:0-22:6 PtdCho, respectively.

Figure 1 describes the identification procedure for 16:0-22:6 PtdCho with [M + Na]^+^ at *m/z* 828.4 in salmon muscle tissue using NL scanning in the positive mode. The confirmation of the phosphocholine group of unknown peak [M + Na]^+^ at *m/z* 828.4 was conducted through NL 183.1. Indeed, the peak of [M + Na]^+^ at *m/z* 828.4 was observed in NL 183.1, which indicates the presence of the phosphocholine moiety in the chemical structure. A wide spectrum of NL scanning was performed to search for the corresponding two aliphatic chains of the unknown molecule. The unknown peak [M + Na]^+^ at *m/z* 828.4 was also found in both NL 256.0 and NL 328.0, accounting for FA 16:0 (palmitic acid) and FA 22:6 (docosahexaenoic acid), respectively, leading to the identification of 16:0-22:6 PtdCho. NL 59.0 was also scanned to verify the existence of a trimethylamine group in 16:0-22:6 PtdCho. In this study, the entire identification process of phospholipids was followed as described in Figure 1.

The list of PCs found in salmon muscle tissue after assembly of the phosphate moiety and two acyl chains based on NL scanning is summarized in Table S1. In this study, 15 PCs were identified in lipid extracts of salmon muscle tissue, and all of them contained at least one unsaturated fatty acid. The dominant PC in the sample was 16:0-22:6 PtdCho [M + Na]^+^ at *m/z* 828.4. Boselli et al. [24] explored phospholipid profiles in several types of fish, such as horse mackerel, European hake, European anchovy, European pilchard, and Atlantic mackerel, and found a high level of 16:0-22:6 PtdCho in all fish tested.

In the present study, most PCs possessed PUFAs such as 20:2, 20:3, 20:4, 20:5, 22:4, 22:5, and 22:6, except 16:0-18:1 PtdCho. According to the literature, salmon muscle tissue contains a high level of PUFAs. In particular, they possess a large percentage of eicosapentaenoic acid (EPA, 20:5) and docosahexaenoic acid (DHA, 22:6), accounting for 6.4%–8.9% and 13.1%–19.4% of the total fatty acids in salmon muscle tissue, respectively [25,26]. This information supports the abundant occurrence of EPA and DHA in PC in the present study. However, there is a limitation in the identification of such PUFA-containing phospholipids in the use of shotgun-based ESI MS/MS since ESI-MS/MS analysis cannot determine double bond positions and therefore the identity of these fatty acids is necessarily inferred.

### 2.2. Identification of PE, PS, and PI in Salmon Muscle Tissue by Precursor Ion Scanning in the Negative Mode

The assemblies of the phosphate moiety and two acyl chains of PE, PS, and PI in salmon muscle tissue after precursor ion scanning are summarized in Table S2. Mass spectrometry analysis of these three classes of phospholipids was performed in the negative mode due to its more efficient ionization of the molecules compared to the positive mode. The glycerol phosphoethanolamine portion of the PE was confirmed through the precursor ion scanning mode at 196.0, followed by the assembly of the other two fatty acids.

In this work, a total of 19 species of PE were found in lipid extracts of salmon muscle tissue, and all of them possessed unsaturated fatty acids. Eicosapentaenoic acid (EPA, 20:5) and docosahexaenoic acid (DHA, 22:6) were also major fatty acids in PE. Compared to PC and PE, relatively smaller numbers of PS and PI species were identified in salmon muscle tissue. The polar head moiety of PS was verified by precursor-ion scanning at 153.0, corresponding to the glycerol phosphate group. Even though precursor-ion scanning at 153.0 can also detect other lipid species, the discrepancy in molecular weight (*m*/*z*) between molecules that are detected with precursor-ion scanning at 153.0 led to the efficient identification process by distinguishing each molecule.

A total of five PS species—16:0-22:6, 18:0-20:5, 18:0-22:6, 18:0-22:5, and 22:5-22:6 PtdSer—were found in the present contribution. All molecules had eicosapentaenoic acid (EPA, 20:5) or docosahexaenoic acid (DHA, 22:6) in their chemical structure.

The identification process of PI was more difficult compared to other classes of phospholipids due to the weak signals, which might be due to the low level of PI in salmon extract or low efficiency in the ionization process. Four PIs—16:0-22:5, 18:0-20:5, 18:0-22:6, and 20:1-20:5 PtdIns—were found in this study.

### 2.3. Quantification of Phospholipids in Salmon Muscle Tissue

The quantification of phospholipids in salmon muscle tissue was conducted using external standards such as 18:1-18:1 phosphatidylcholine, 18:1-18:1 phosphatidylethanolamine, 16:0-16:0 phosphatidylserine, and 18:1-18:1 phosphatidylinositol, which are the representative molecules of PC, PE, PS, and PI, respectively. In this work, the major variables, such as the solvent for dissolving the lipid extract before injection, the concentration of sodium hydroxide to induce the formation of adducts, and the optimization of the ESI-MS-MS condition, were focused on improving the accuracy of the quantification.

**Figure 2:**
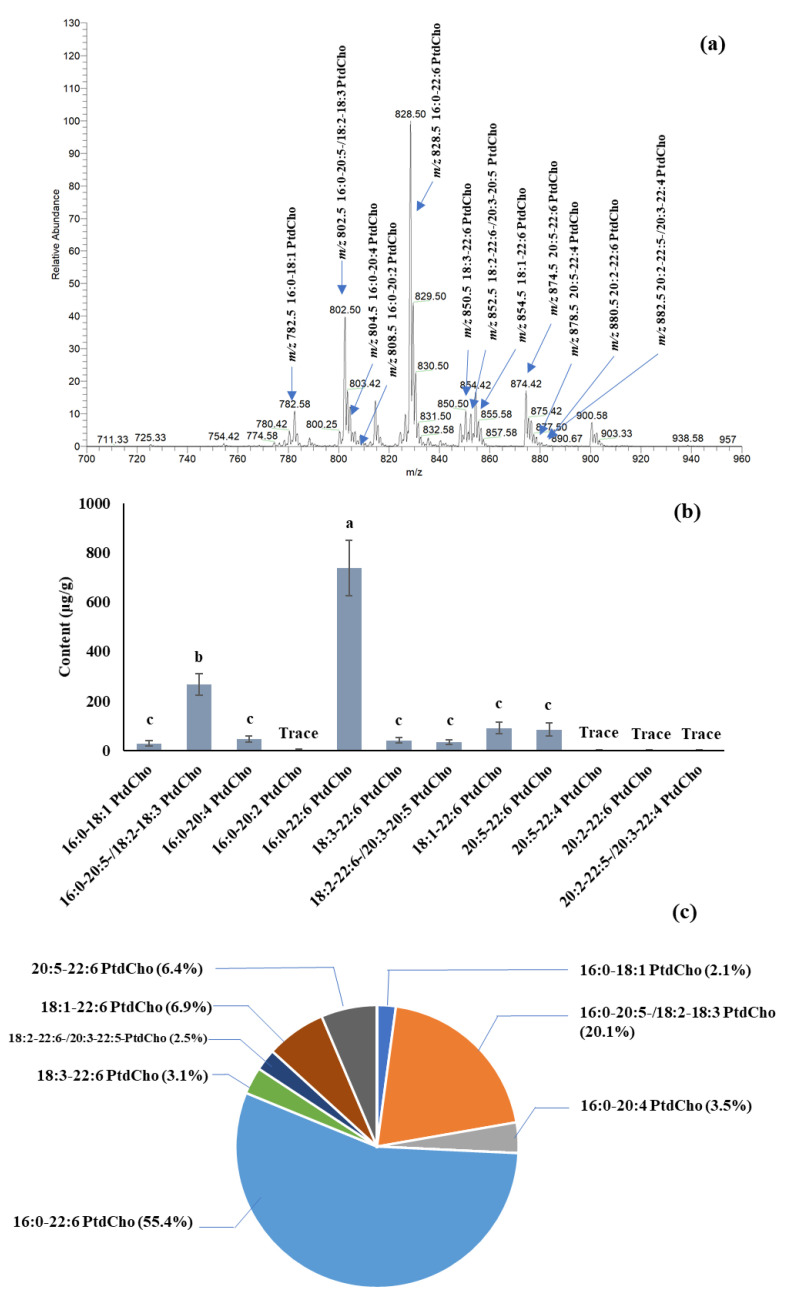
Identification and quantification of phosphatidylcholines (PCs) in salmon muscle tissue. (**a**) Mass spectrum at NL 183.1 and identified PC species in salmon muscle tissue; (**b**) quantification of individual PCs ^1^ (error bars presented in Figure 2b indicate standard deviation calculated from three independent replicates); (**c**) proportion of each PC species in salmon muscle tissue. ^1^ Values having the same letter are not significantly different (*p* > 0.05). The different letters (i.e., a, b, c, etc.) indicate significant differences among values, and the largest value is denoted as “a”.

The quantification of PC species in salmon muscle tissue is summarized in Figure 2. The dominant PC was 16:0-22:6 PtdCho [M + Na]^+^ at *m/z* 828.4, among others, with amounts of 738.0 μg/g in fresh tissue. Other PCs, such as 16:0-20:5-, 18:2-18:3-, 18:1-22:6-, and 20:5-22:6-phosphatidylcholine, were also the major PCs in salmon muscle tissue. The quantification of PE, PS, and PI species in salmon muscle tissue is summarized in Table 1. A comparable amount of PEs was found in salmon muscle tissue. The PE content ranged from 35.7 to 92.0 μg/g, except for 18:3-20:5-,18:2-20:5-, and 20:2-22:6-phosphatidylethanolamine, of which only trace amounts were detected. A [M + Na]^+^ peak at *m/z* 762.6, which is the combination of 16:0-22:6-, 18:1-20:5-, and 18:2-20:4-phosphatidylethanolamine, displayed the highest intensity among others. Table 1 also summarizes the level of PSs and PIs in salmon muscle tissue. PSs and PIs showed lower levels compared to the PCs and PEs. Five different PS species, substituted with 16:0-22:6, 18:0-20:5, 18:0-22:6, 18:0-22:5, and 22:5-22:6, were found in the lipid extract of salmon muscle tissue, and the range of their contents was 10.1–27.9 μg/g. In addition, four PIs, having 16:0-22:5-, 18:0-20:5, 18:0-22:6, and 20:1-20:5 fatty acid combinations, were identified, with a total of 7.3 μg/g. A total of 1210.7, 593.7, 77.5, and 7.5 μg/g of PC, PE, PS, and PI were quantified in the salmon muscle tissue, and PC and PE were the main phospholipid classes compared to PS and PI.

Several studies have been conducted on the determination of phospholipid profiles in salmon muscle tissue. Song et al. [27] identified 37 phospholipid species in domestic rainbow trout and Atlantic and king salmon using rapid evaporative ionization mass spectrometry. Boselli et al. [24] also identified a variety of phospholipid species in bony fish and shellfish using HPLC-MS/MS. However, these studies only provided the proportion/percentage of each phospholipid species by using the ion intensity of each compound in MS/MS analysis, but they did not conduct the quantification of individual phospholipid species by using internal or external standards. Thus, the quantification of individual phospholipids in the present study provides useful information for understanding lipidomics in salmon.

### 2.4. Identification and Quantification of Eicosanoids in Salmon Muscle Tissue

**Figure 3:**
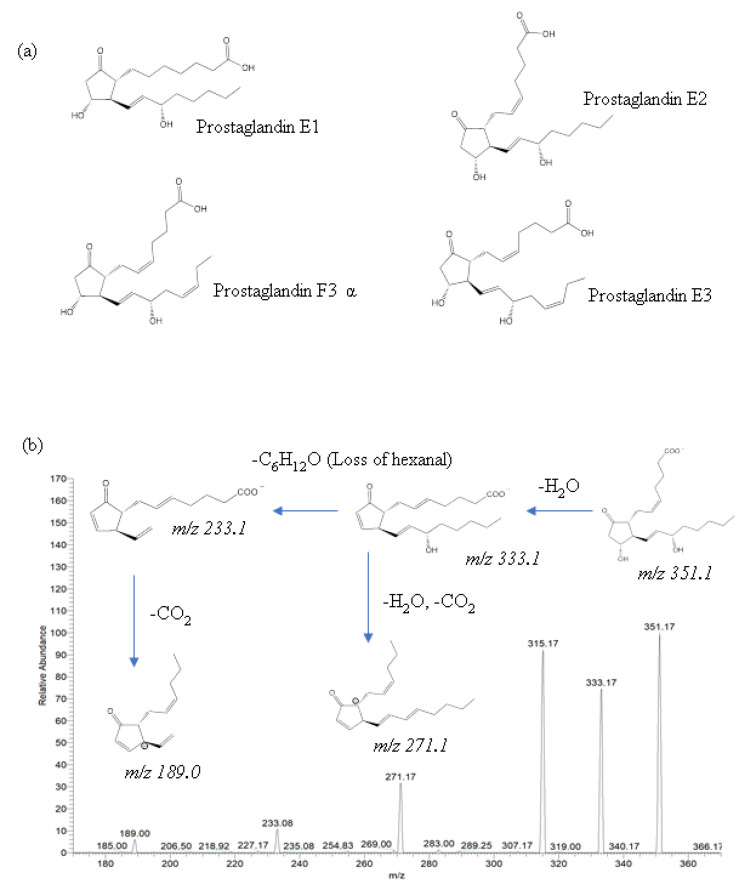
Chemical structure of four eicosanoids tested (**a**) and MS/MS spectrum of PGE2 showing its fragmentation pathway in the negative ion mode (**b**).

The identification of eicosanoids in salmon muscle tissue was performed using ESI-MS/MS analysis by comparing fragment and precursor ions with corresponding standard compounds, including prostaglandin E1 (PGE1), prostaglandin E2 (PGE2), prostaglandin E3 (PGE3), and prostaglandin F3α (PGF3α) (Figure 3a), and the quantification of eicosanoids was calculated using external calibration.

In the present study, two eicosanoids—PGE2 and PGF3α—were identified in salmon muscle tissue, whereas PGE1 and PGE3 were not identified due to the mismatch in product ions. The major product ions of PGE2 were 189.0, 233.1, 271.1, 315.1, and 333.1, and their fragmentation pathways are described in Figure 3b. In the lipid extract of salmon muscle tissue, a deprotonated molecule [M–H]^−^ at *m*/*z* 351.1 was detected, which displays the same product ions as PGE2; thus, this molecule was identified as being a PGE2. Moreover, the quantitative analysis showed the presence of 16.2 ± 3.0 ng/g PGE2 in the salmon muscle tissue (Table 2). According to the literature, PGE2 has been found in a mouse model. Brose et al. [28] identified PGE2 in mouse brain tissue using LC-MS/MS, and the level of PGE2 was 15.3 ng/g upon decapitation-induced ischemia, which is a similar level to that of the present study (salmon muscle tissue). PGE2 was also found in human plasma, and the range of PGE2 was 1–29 pg/mL of plasma [29,30]. Oxley et al. [31] reported that Atlantic salmon intestinal tissue contained approximately 10–30 ng/g of PGE2. However, PGE2 has not been found in Atlantic salmon muscle; thus, the present study reports the identification and quantification of PGE2 in salmon muscle tissue by ESI-MS/MS for the first time.

In addition to PGE2, PGF3α was also identified for the first time in salmon muscle tissue. A deprotonated molecule [M–H]^−^ at *m*/*z* 351.1, along with product ions such as 247.1, 192.1, 307.1, 194.1, were detected in the lipid extract of salmon muscle tissue, and the quantitative analysis showed the presence of 31.1 ± 0.7 μg/g in the salmon muscle tissue, which is a higher value compared to PGE2.

## 3. Discussion

**Figure 4:**
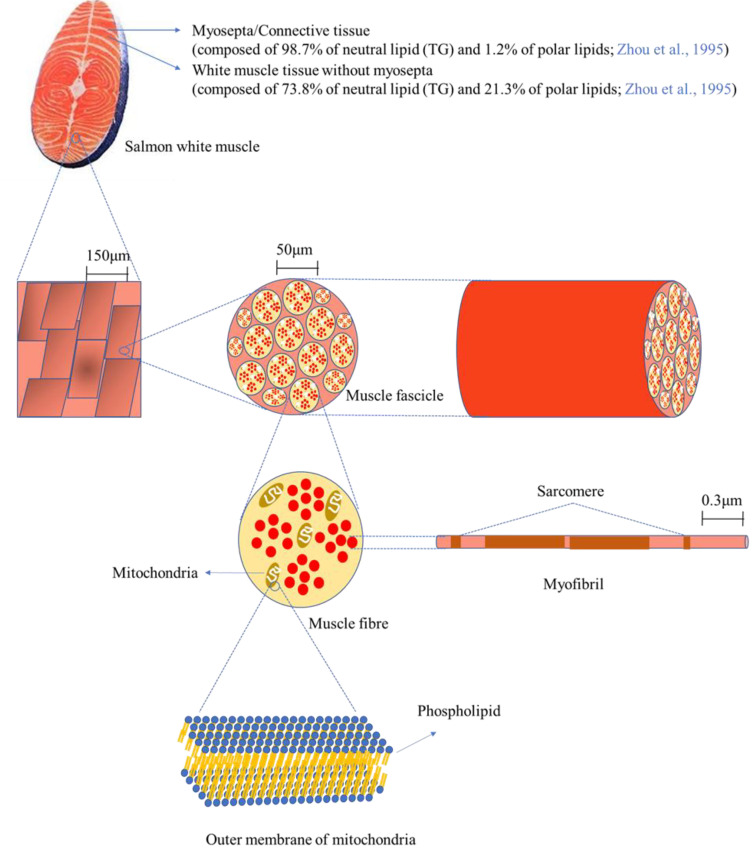
The microstructure of the white muscle tissue of salmon and the localization of phospholipids in the outer membrane of mitochondria (other organelles such as the nucleus are not shown).

Different fish species have different compositions of phospholipids, and this discrepancy affects the functions of phospholipids such as the fluidity of membranes and their performance as signalling molecules. Figure 4 depicts the microstructure of white muscle tissue of salmon at different magnifications. Salmon tissue mainly consists of white and red/dark muscle tissue (not shown in the figure), along with myosepta. The white line of myosepta stores a large amount of lipid/fat in salmon tissue, accounting for 39.1% of white tissue and 62.4% of red/dark tissue [32]. In this study, only white tissue was used for the mass spectrometry analysis after separation from red/dark tissue; thus, white tissue will be focused on here. The myosepta in white tissue is composed of 98.7% neutral lipids (TAG) and 1.2% polar lipids (mainly phospholipids), whereas white muscle tissue without myosepta consists of 73.8% neutral lipids (TAG) and 21.3% polar lipids, indicating that phospholipids are primarily localized in white muscle tissue without myosepta [32]. Scanning electron microscope (SEM, 200×) images display a magnified surface of white tissue, and a higher resolution (10,000×) allows observation of more elaborate structures such as muscle fascicles, which are bundles of muscle fibres (cells) [33]. Muscle fibre cells contain various organelles such as the nucleus, mitochondria, and endoplasmic reticulum, as well as myofibrils, which are responsible for muscle contraction and relaxation. Phospholipids are the main building materials for the construction of the lipid-bilayer membrane, providing a barrier from the extracellular space as well as between organelles. Since the lipid-bilayer membrane is found in most of the aforementioned organelles, the composition of phospholipids, in particular the carbon chain length and the number of double bonds, greatly influences the function of the cells. In the present work, most of the phospholipids contained PUFAs such as 20:2, 20:3, 20:5, 22:4, 22:5, and 22:6. The fatty acid compositions of phospholipids affect the fluidity of the membrane, thus it is expected that the high unsaturation of the fatty acids in the phospholipids may enhance the fluidity of the membrane in salmon tissue, especially by DHA (22:6) and EPA (20:5).

Compared to EPA and DHA, linolenic acid (18:3) was a minor component as a constituent of PC; namely, only two PCs: 18:2-18:3 PtdCho and 18:3-22:6 PtdCho were found to possess linolenic acid. Different types of salmon, including chum, coho, cherry, and Atlantic salmon, can use linolenic acid (18:3ω3) as the essential ω3 fatty acid for their metabolism, especially at the juvenile and sub-adult stage, and the level of requirement is 1.0% of the dry diet [34,35]. The ingested linolenic acid is mainly used as the energy source for cellular metabolism, as well as a precursor for EPA and then DHA synthesis, and this might be the reason for the low occurrence of linoleic acid in PC in this study [36]. Conversely, DHA is not a suitable substrate for the β-oxidation process to generate energy, leading to its accumulation in salmon tissue [37]. This partially explains the high level of DHA in salmon tissue as shown in this work. In summary, shotgun-based mass spectrometric analysis enabled the effective identification and quantification of phospholipids in salmon muscle tissue. The developed shotgun-based mass spectrometric analysis and its combination with HPLC (or other equipment such as supercritical fluid chromatography) could be applied to a wide range of research areas, including lipidomics in natural sources and other fields such as nutrition, drug discovery and screening, and human health (i.e., cancer, neurological disorder, eye disease, etc.) [38].

In the present study, two eicosanoids—PGE2 and PGF3α—were identified and quantified in salmon muscle tissue. The difference in the levels of PGE2 and PGF3α in salmon muscle tissue might be due to the discrepancy in the levels of precursor molecules required for the synthesis of those two compounds. In other words, salmon muscle tissue contains 0.3%–0.7% of 20:4 ω6 and 1.3%–3.2% of 20:5 ω3, which are the precursor molecules for the synthesis of PGE2 and PGF3α, respectively [39]. Other growth conditions such as the composition of diets, temperature, and other factors can also affect the synthesis of eicosanoids in salmon muscle tissue. Bell et al. [40] reported that 1.3–2.0 ng/g of PGF3α was found in the kidney of Atlantic salmon using HPLC. However, PGF3α has not been identified and quantified in muscle tissue of Atlantic salmon, thus the present study provides useful data for investigating PGF3α in salmon muscle. Shotgun-based mass spectrometric analysis enabled the effective identification and quantification of PGE2 and PGF3α in salmon muscle tissue. However, given the possibility of the presence of other isobaric compounds having the same fragmentation pathway as PGE2 and PGF3α, which interferes with the quantitative analysis, the association of this approach with HPLC would improve the accuracy of identification and quantification of PGE2 and PGF3α.

## 4. Materials and Methods

### 4.1. Materials

Atlantic salmon (*Salmo salar* L.) was purchased at a local market. Standards such as 18:1-18:1 phosphatidylcholine, 18:1-18:1 phosphatidylethanolamine, 18:1-18:1 phosphatidylinositol, and 16:0-16:0 phosphatidylserine were purchased from Sigma-Aldrich Canada Ltd. (Oakville, ON, Canada). Eicosanoid standards such as EPA Oxylipin mixture, containing prostaglandin E3, prostaglandin F3α, and a mixture of prostaglandin E2 and PGD E1, were obtained from Cayman Chemical Company (Ann Arbor, MI, United States). Sodium hydroxide, methanol (HPLC grade), and chloroform (HPLC grade) were purchased from Fisher Scientific Co (Nepean, ON, Canada).

### 4.2. Extraction of Crude Lipids from Salmon Muscle Tissue

The extraction of lipophilic compounds in salmon muscle tissue was carried out as described by Parrish [41], as used routinely in aquaculture work [42,43,44,45]. Salmon muscle tissue (250 mg) was mixed with 2 mL of ice-cold chloroform and 1 mL of methanol in a 15-mL glass vial. The mixture was homogenized using a blender (Polytron Homogenizer PT 3300, Kinematica AG, Luzern, Switzerland) with a metal rod, and the rod was subsequently washed with 1 mL of chloroform:methanol (2:1) and 0.5 mL of chloroform-extracted water. The samples were sonicated (300Ultrasonik, Whittemore Enterprises, Inc., Rancho Cucamonga, CA, USA) for four min in an ice bath, followed by centrifugation at 3500 *g* (IEC Centra MP4, International Equipment Co., Needham Heights, MA, USA) for two min. The organic layer (bottom) containing lipophilic compounds was isolated/transferred to a clean glass vial using a double pipetting technique; namely, a 2-mL Pasteur pipette was placed inside a 1-mL pipette to prevent the disruption of the aqueous layer. The entire procedure was repeated three times, and all organic portions were combined into a lipid-clean vial. The organic solvent in the vials was removed through nitrogen evaporation, and the lipid was re-dissolved with 1 mL of chloroform. The final solution was kept at −20 ℃ under nitrogen until further use.

### 4.3. ESI-MS/MS Analysis for the Determination of Phospholipid Profiles

The identification of phospholipids in salmon muscle tissue was performed using a triple quadrupole mass spectrometer (TSQ Quantis™ Triple Quadrupole Mass Spectrometer, Thermo Fisher Scientific, Waltham, MA, USA) equipped with a mass selective detector (MSD) ion trap system after electrospray ionization (ESI). The drying gas (N_2_) temperature was fixed at 350 °C. The lipid extract (100 μL) of salmon muscle tissue was diluted with 890 μL of 1:4 chloroform:methanol (*v*/*v*) for the preparation of 25 mg/mL of the final solution, followed by the addition of 10 μL of sodium hydroxide (final concentration) to induce sodium adduct formation with the phospholipids. Five hundred microlitres of the sample solution were injected into the mass spectrometer (direct infusion) at a flow of 10 μL/min using a high-pressure syringe pump (F100T2, Chemyx Inc., Stafford, TX, USA). The identification of PC was conducted in the positive mode, with 4000 (v) ion spray voltage, 50 (arb) sheath gas, 10 (arb) aux gas, 1 (arb) sweep gas, 325 °C ion transfer tube temperature, and 30 °C vaporizer temperature. The confirmation of the hydrophilic head portion and two fatty acids of PCs was performed using the neutral loss scanning mode. To confirm the presence of the phosphate group, the neutral loss was set at 183.1, and the specific neutral losses used for fatty acyl chains are provided in Table S1. In the neutral loss, the scan rate was fixed into 1000 DA/sec and 200 times scanning was carried out for each scanning. The collision energy was set at 37 eV, and the CID gas was fixed at 1 mTorr. The identification of other phospholipids such as PE, PS, and PI was conducted in the negative mode with 2500 (v) ion spray voltage, 2.0 (arb) sheath gas, 13.8 (arb) aux gas, 0.8 (arb) sweep gas, 325 °C ion transfer tube temperature, and 30 °C vaporizer temperature. The same collision energy and CID gas were applied in the precursor-ion scanning mode as used in the positive mode. The same scan rates and scanning times were used in the precursor-ion scanning mode, and the detailed precursor-ion scanning conditions for each class of PLs are given in Table S2.

### 4.4. Quantification of Phospholipids in Salmon Muscle Tissue

For triplicate measurements, the extraction of crude lipids in salmon muscle tissue was conducted on three different days as described in the extraction section. The quantification of phospholipids in salmon muscle tissue was conducted using external standards such as 18:1-18:1 phosphatidylcholine, 18:1-18:1 phosphatidylethanolamine, 16:0-16:0 phosphatidylserine, and 18:1-18:1 phosphatidylinositol as the representative PC, PE, PS, and PI, respectively. A standard curve of 1,2-dioleoyl-*sn*-glycero-3-phosphocholine was made with NL 183.1 in the positive ion mode, and the standard curve equation *y* = 7423.5*x* + 10034 (*y*: intensity, *x*: concentration of standard) was used for the quantification of PCs in salmon muscle tissue. The level of PE, PS, and PI were also calculated in a similar way using precursor-ion scanning at 196.0, 153.0, and 241.0, respectively, in the negative ion mode. The equations from standard curves of 18:1-18:1 phosphatidylethanolamine, 16:0-16:0 phosphatidylserine, and 18:1-18:1 phosphatidylinositol at precursor-ion scanning 196.0, 153.0, and 241.0 were *y*=15.77*x*−8.125, *y*=24.86*x*−6.858, and *y*=103.9*x*+13.33, respectively, and these were applied for quantitative analysis of PE, PS, and PI in the salmon muscle tissue.

### 4.5. Optimization of Sodium Hydroxide Inclusion to Produce Sodium Adducts of Phospholipids

A sodium hydroxide solution in methanol was prepared to investigate the optimal concentration of sodium hydroxide for the production of phospholipid sodium adducts. Different concentrations of sodium hydroxides (i.e., 0.1, 0.5, 1.0, and 10.0 mM) were added to the lipid extract of salmon muscle tissue, followed by injection into the ESI mass spectrometer (Figure S1). The resulting intensities in the mass spectra were compared to seek the optimal concentration of sodium hydroxide inclusion for the identification and quantification of phospholipids in salmon muscle tissue.

### 4.6. ESI-MS/MS Analysis for the Identification and Quantification of Eicosanoids

First, MS/MS parameters were optimized to acquire high selectivity for each standard compound. The identification of PGE2 and PGF3α was carried out in the negative mode with 4490 and 4363 (v) ion spray voltage, 4.0 and 2.4 (arb) sheath gas, 6.1 and 20.7 (arb) aux gas, 7.1 and 0.4 (arb) sweep gas, 18.26 and 10.23 eV of collision energy, respectively. The same ion transfer tube temperature (325 °C), vaporizer temperature (30 °C), and CID gas (1 mTorr) were set for the analysis of both compounds. The quantification of eicosanoids was carried out using external standards.

### 4.7. Statistical Analysis

Three independent replicates were used for conducting statistical analyses, the data were used for one-way ANOVA, and each mean was compared using Tukey’s HSD test (*p* < 0.05) in SPSS 16.0 for Windows (SPSS Inc., Chicago, IL, USA). Error bars presented in Figure 2b indicate standard deviation calculated from three independent replicates.

## 5. Conclusions

The identification and quantification of phospholipids in salmon muscle tissue were performed using ESI-MS-MS, equipped with an MSD ion trap system. A total of 43 species of phospholipids were identified by assembling the phosphate group and two fatty acid moieties after neutral loss or precursor-ion scanning. ESI-mass spectrometry showed high performance in detecting ions derived from phospholipids through the NL and precursor-ion mode. A relatively lower number of PSs and PIs was identified in salmon muscle tissue compared to the PCs and PEs. This is, to the best of our knowledge, the first quantification of individual phospholipids in salmon muscle tissue. In addition, two eicosanoids—PGE2 and PGF3α—were identified and quantified in salmon muscle tissue for the first time. Nowadays, the application of HPLC systems for the separation of individual compounds in the samples prior to mass spectrometric analysis has been widely used for the analysis of PLs and eicosanoids, along with the use of internal standard analysis for quantification. This approach may improve the accuracy of the identification and quantification analysis of PLs and eicosanoids in samples; thus, the application of HPLC systems to mass spectrometric analysis in future work could be a useful strategy for PL and eicosanoid analysis in biological samples. In addition, the use of an accelerated solvent extractor, which is a rapid and high-throughput strategy to avoid cross contamination in the extraction process, should be investigated in order to supplement the extraction method used in the present study.

## Figures and Tables

**Table 1:** Quantification of phosphatidylethanolamines (PEs), phosphatidylserines (PSs), and phosphatidylinositols (PIs) in salmon muscle tissue.^1.^

[M–H]^-^	PE	Quantification (μg/g)
736.7	16:0-20:5 PtdEtn	40.0 ± 15.3c
758.4	18:3-20:5 PtdEtn	Trace
760.6	18:2-20:5 PtdEtn	Trace
762.6	16:0-22:6 PtdEtn/ 18:1-20:5 PtdEtn/ 18:2-20:4 PtdEtn	92.0 ± 17.1a
764.0	16:0-22:5 PtdEtn/ 18:0-20:5 PtdEtn/ 18:1-20:4 PtdEtn	60.3 ± 17.9abc
786.6	18:2-22:6 PtdEtn	43.5 ± 5.0c
788.3	18:2-22:5 PtdEtn	84.0 ± 15.4ab
790.0	18:0-22:6 PtdEtn/ 18:1-22:5 PtdEtn	41.1.5 ± 7.5c
808.8	20:5-22:6 PtdEtn	49.5 ± 12.2bc
812.9	20:3-22:6 PtdEtn	44.6 ± 3.2bc
814.8	20:2-22:6 PtdEtn	Trace
834.7	22:6-22:6 PtdEtn	56.2 ± 21.7abc
836.0	22:5-22:6 PtdEtn	46.8 ± 13.9bc
838.6	22:4-22:6 PtdEtn	35.7 ± 9.3c
**[M–H]^-^**	**PS**	**Quantification (μg/g)**
806.1	16:0-22:6 PtdSer	15.1 ± 4.9b
808.1	18:0-20:5 PtdSer	10.1 ± 0.1b
834.1	18:0-22:6 PtdSer	13.6 ± 4.6b
836.4	18:0-22:5 PtdSer	10.8 ± 0.2b
880.0	22:5-22:6 PtdSer	27.9 ± 3.9a
**[M–H]^-^**	**PI**	**Quantification (μg/g)**
883.1	16:0-22:5 PtdIns/ 18:0-20:5 PtdIns	2.1 ± 2.5a
909.8	18:0-22:6 PtdIns/ 20:1-20:5 PtdIns	5.4 ± 1.6a

^1^ Mean ± standard deviation; statistical analysis was carried out for all three groups—PE, PS, and PI—and it was conducted within the same PL group; values in each column within the same PL class having the same letter are not significantly different (*p* > 0.05). The difference in letters (i.e., a, b, c, etc.) indicates a significant difference among values, and the largest value is denoted as “a”.

**Table 2:** Identification and quantification of eicosanoids in salmon muscle tissue.

[M–H]^-^	Eicosanoid	Quantification	Product Ions
351.1	PGE2	16.2 ± 3.0 ng/g	271, 315, 189, 333
351.1	PGF3α	31.1 ± 0.7 μg/g	247, 192, 307, 194
353.1	PGE1	ND	317, 273, 309, 235
349.1	PGE3	ND	269, 313, 261, 331

ND: Not detected.

## Data Availability

The data presented in this study will be openly available.

## Supplementary Materials

The following are available online at https://www.mdpi.com/1422-0067/22/5/2272/s1, Table S1. List of identified phosphatidylcholines in salmon tissue in the positive ion mode, Table S2. List of identified PE, PS, and PI in salmon tissue in the negative ion mode using the precursor ion (PI) scanning mode, Figure S1. Precursor ion (PI) scanning (196.0) of a salmon extract at different concentrations of sodium hydroxide addition in order to optimize the ionization of phospholipids in the negative mode.
