# Supplementary material for: Shotgun Lipidomics for the Determination of Phospholipid and Eicosanoid Profiles in Atlantic Salmon (Salmo salar L.) Muscle Tissue Using Electrospray Ionization (ESI)-MS/MS Spectrometric Analysis
Source: Int J Mol Sci. 2021 Feb 25;22(5):2272. doi: 10.3390/ijms22052272 (PMC7956627; doi:10.3390/ijms22052272)
Supplement: Supplementary file 1 [file ijms-22-02272-s001.pdf]

## Supplementary Data

**Table S1. List of identified phosphatidylcholines in salmon tissue in the positive ion mode**

| | | MS/MS scanning | | |
|---|---|---|---|---|
| **[M + Na]$^+$** | **Phosphatidylcholine** | **Polar head group** | **Fatty acid 1** | **Fatty acid 2** |
| 782.4 | 16:0-18:1 PtdCho | NL 183.1 (phosphocholine) | NL 256.0 (16:0) | NL 282.0 (18:1) |
| 802.4 | 16:0-20:5 PtdCho | NL 183.1 (phosphocholine) | NL 256.0 (16:0) | NL 302.0 (20:5) |
| 802.4 | 18:2-18:3 PtdCho | NL 183.1 (phosphocholine) | NL 280.0 (18:2) | NL 278.0 (18:3) |
| 804.5 | 16:0-20:4 PtdCho | NL 183.1 (phosphocholine) | NL 256.0 (16:0) | NL 304.0 (20:4) |
| 808.4 | 16:0-20:2 PtdCho | NL 183.1 (phosphocholine) | NL 256.0 (16:0) | NL 308.0 (20:2) |
| 828.4 | 16:0-22:6 PtdCho | NL 183.1 (phosphocholine) | NL 256.0 (16:0) | NL 328.0 (22:6) |
| 850.4 | 18:3-22:6 PtdCho | NL 183.1 (phosphocholine) | NL 278.0 (18:3) | NL 328.0 (22:6) |
| 852.4 | 18:2-22:6 PtdCho | NL 183.1 (phosphocholine) | NL 280.0 (18:2) | NL 328.0 (22:6) |
| 852.4 | 20:3-20:5 PtdCho | NL 183.1 (phosphocholine) | NL 306.0 (20:3) | NL 302.0 (20:5) |
| 854.4 | 18:1-22:6 PtdCho | NL 183.1 (phosphocholine) | NL 282.0 (18:1) | NL 328.0 (22:6) |
| 874.4 | 20:5-22:6 PtdCho | NL 183.1 (phosphocholine) | NL 302.0 (20:5) | NL328.0 (22:6) |
| 878.5 | 20:5-22:4 PtdCho | NL 183.1 (phosphocholine) | NL 302.0 (20:5) | NL 332.0 (22:4) |
| 880.5 | 20:2-22:6 PtdCho | NL 183.1 (phosphocholine) | NL 308.0 (20:2) | NL 328.0 (22:6) |
| 882.5 | 20:2-22:5 PtdCho | NL 183.1 (phosphocholine) | NL 308.0 (20:2) | NL 330.0 (22:5) |
| 882.5 | 20:3-22:4 PtdCho | NL 183.1 (phosphocholine) | NL 306.0 (20:3) | NL 332.0 (22:4) |

**Table S2. List of identified PE, PS, and PI in salmon tissue in the negative ion mode using the precursor ion (PI) scanning mode**

| $[M - H]^-$ | PE | MSMS scanning | | |
|---|---|---|---|---|
| | | **Polar head group** | **Fatty acid 1** | **Fatty acid 2** |
| 736.7 | 16:0-20:5 PtdEtn | PI 196.0 (glycerol phosphoethanolamine) | PI 255.0 (16:0) | PI 301.0 (20:5) |
| 758.4 | 18:3-20:5 PtdEtn | PI 196.0 | PI 277.0 (18:3) | PI 301.0 (20:5) |
| 760.6 | 18:2-20:5 PtdEtn | PI 196.0 | PI 279.0 (18:2) | PI 301.0 (20:5) |
| 762.6 | 16:0-22:6 PtdEtn | PI 196.0 | PI 255.0 (16:0) | PI 327.0 (22:6) |
| 762.6 | 18:1-20:5 PtdEtn | PI 196.0 | PI 281.0 (18:1) | PI 301.0 (20:5) |
| 762.6 | 18:2-20:4 PtdEtn | PI 196.0 | PI 279.0 (18:2) | PI 303.0 (20:4) |
| 764.0 | 16:0-22:5 PtdEtn | PI 196.0 | PI 255.0 (16:0) | PI 329.0 (22:5) |
| 764.0 | 18:0-20:5 PtdEtn | PI 196.0 | PI 283.0 (18:0) | PI 301.0 (20:5) |
| 764.0 | 18:1-20:4 PtdEtn | PI 196.0 | PI 281.0 (18:1) | PI 303.0 (20:4) |
| 786.6 | 18:2-22:6 PtdEtn | PI 196.0 | PI 279.0 (18:2) | PI 327.0 (22:6) |
| 788.3 | 18:2-22:5 PtdEtn | PI 196.0 | PI 279.0 (18:2) | PI 329.0 (22:5) |
| 790.0 | 18:0-22:6 PtdEtn | PI 196.0 | PI 283.0 (18:0) | PI 327.0 (22:6) |
| 790.0 | 18:1-22:5 PtdEtn | PI 196.0 | PI 281.0 (18:1) | PI 329.0 (22:5) |
| 808.8 | 20:5-22:6 PtdEtn | PI 196.0 | PI 301.0 (20:5) | PI 327.0 (22:6) |
| 812.9 | 20:3-22:6 PtdEtn | PI 196.0 | PI 305.0 (20:3) | PI 327.0 (22:6) |
| 814.8 | 20:2-22:6 PtdEtn | PI 196.0 | PI 307.0 (20:2) | PI 327.0 (22:6) |
| 834.7 | 22:6-22:6 PtdEtn | PI 196.0 | PI 327.0 (22:6) | PI 327.0 (22:6) |
| 836.0 | 22:5-22:6 PtdEtn | PI 196.0 | PI 329.0 (22:5) | PI 327.0 (22:6) |
| 838.6 | 22:4-22:6 PtdEtn | PI 196.0 | PI 331.0 (22:4) | PI 327.0 (22:6) |
| **$[M - H]^-$** | **PS** | | | |
| 806.1 | 16:0-22:6 PtdSer | PI 153.0 (glycerol phosphate derivative) | PI 255.0 (16:0) | PI 327.0 (22:6) |
| 808.1 | 18:0-20:5 PtdSer | PI 153.0 | PI 283.0 (18:0) | PI 301.0 (20:5) |
| 834.1 | 18:0-22:6 PtdSer | PI 153.0 | PI 283.0 (18:0) | PI 327.0 (22:6) |
| 836.4 | 18:0-22:5 PtdSer | PI 153.0 | PI 283.0 (18:0) | PI 329.0 (22:5) |
| 880.0 | 22:5-22:6 PtdSer | PI 153.0 | PI 329.0 (22:5) | PI 327.0 (22:6) |
| **$[M - H]^-$** | **PI** | | | |
| 883.1 | 16:0-22:5 PtdIns | PI 241.0 (cyclic inositol phosphate) | PI 255.0 (16:0) | PI 329.0 (22:5) |
| 883.1 | 18:0-20:5 PtdIns | PI 241.0 | PI 283.0 (18:0) | PI 301.0 (20:5) |
| 909.8 | 18:0-22:6 PtdIns | PI 241.0 | PI 283.0 (18:0) | PI 327.0 (22:6) |
| 909.8 | 20:1-20:5 PtdIns | PI 241.0 | PI 309.0 (20:1) | PI 301.0 (20:5) |

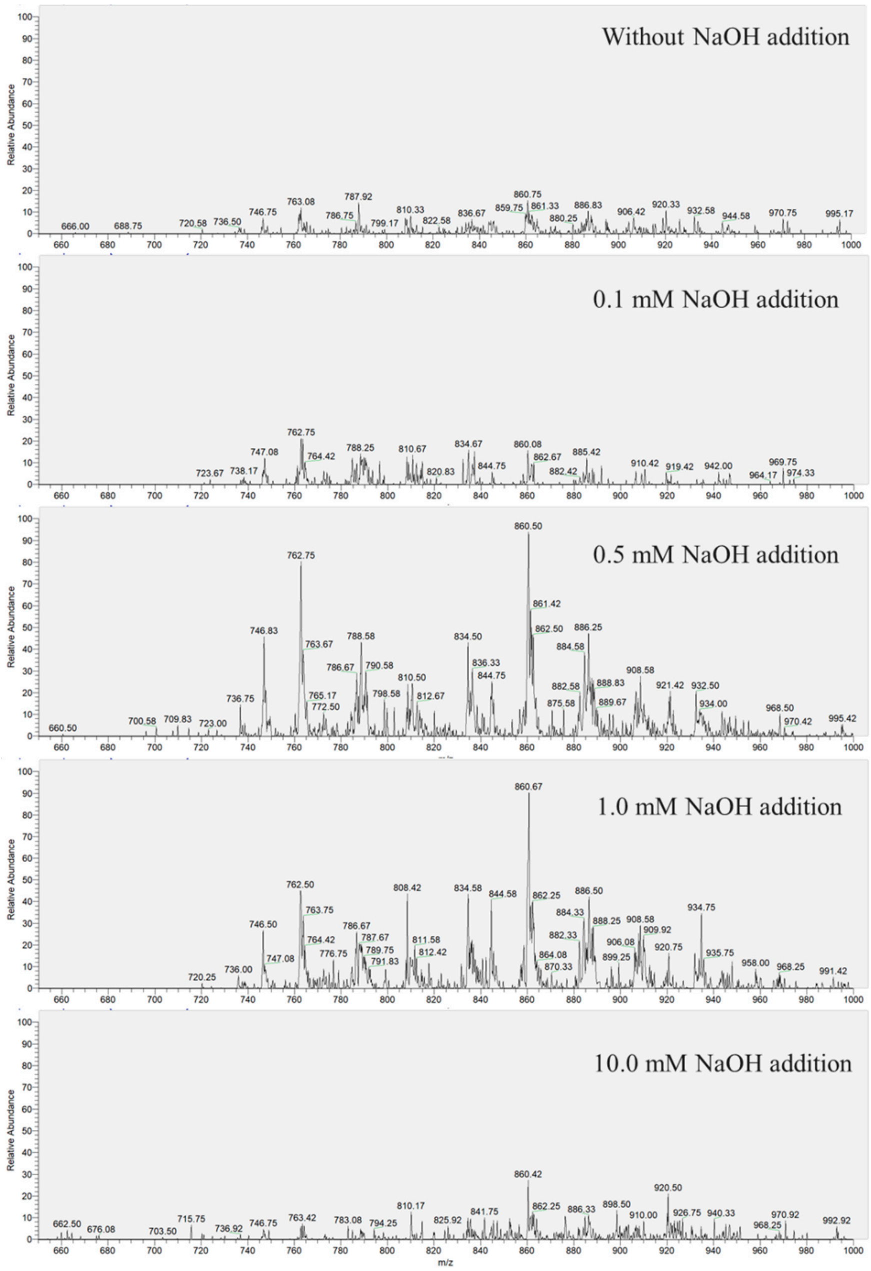


**Figure S1. Precursor ion (PI) scanning (196.0) of a salmon extract at different concentrations of sodium hydroxide addition in order to optimize the ionization of phospholipids in the negative mode.**
